# Real-world outcomes of women with metastatic breast cancer in visceral crisis treated with chemotherapy: a 15-year cohort from Brazil

**DOI:** 10.3332/ecancer.2025.1894

**Published:** 2025-04-17

**Authors:** Matheus de Oliveira Andrade, Vitor Hugo Felix, Laura Testa, Renata Colombo Bonadioa

**Affiliations:** Instituto do Câncer do Estado de São Paulo (ICESP), Faculdade de Medicina da Universidade de São Paulo (FMUSP), São Paulo 01246-000, Brazil; a https://orcid.org/0000-0001-5818-922X

**Keywords:** metastatic breast cancer, visceral crisis, chemotherapy

## Abstract

**Introduction:**

Visceral crisis (VC) in metastatic breast cancer (MBC) is defined as a severe organ dysfunction related to metastatic disease. The cornerstone of treatment for VC relies on polychemotherapy, particularly in low- and middle-income countries, where it often represents the only available therapeutic option. This study aims to assess survival outcomes of palliative chemotherapy (CT) for VC in a real-world scenario.

**Methods:**

Data were retrospectively collected from patients with MBC diagnosed with VC between 2008 and 2022 in a large cancer center in Brazil. Survival analyses were performed using the Kaplan–Meier method. Prognostic factors were evaluated through univariate and multivariable analyses using the Cox regression model.

**Results:**

A total of 146 patients with VC were included. The predominant type of VC was pulmonary (36.3%), hepatic (32.2%) and bone marrow infiltration (19.2%). VC management was based on combination CT (51.4%), while 27.4% were treated with monochemotherapy and 20.6% received best supportive care (BSC). The median overall survival (mOS) for the entire population was 2.17 months. Treatment for VC in the first-line setting was associated with a mOS of 5 months. In the multivariate analysis, hepatic VC and the absence of active oncological therapy (BSC) were significantly associated with mortality.

**Conclusion:**

Patients with MBC in VC have a poor prognosis even when treated with polychemotherapy. Proper prognostication is crucial for identifying patients who may benefit from active systemic therapy while carefully avoiding potentially futile strategies. Prospective trials including patients with VC criteria are needed to evaluate the efficacy and safety of CT and other emerging therapies in this scenario.

## Introduction

Breast cancer (BC) is the leading malignancy in women worldwide and the main cause of cancer-related mortality in this population. Up to nearly one-third of cases will present with metastatic disease in the course of their diagnosis, which may evolve into a critical scenario when there is organ dysfunction secondary to neoplastic dissemination to vital organs, a condition known as visceral crisis (VC) [1–3].

The most recent International Consensus Guidelines for the Management of Advanced Breast Cancer (ABC guidelines 6 and 7) define a VC as ‘severe organ dysfunction as assessed by signs, symptoms, and laboratory studies, resulting from rapid progression of neoplastic disease and indicative of substantial visceral compromise, which may serve as an indication for more aggressive therapeutic intervention.’ However, there are no objective criteria for defining VC. This guideline provides more specific details only for hepatic VC (elevated bilirubin >1.5 times the upper limit of normal in the absence of obstruction) and pulmonary VC (resting dyspnea in the absence of pleural effusion) [4].

Nonetheless, other scenarios have been classified as VC in different studies, based on the clinical perception that the aggressive disease presentation may preclude further treatment if initial management fails. These clinical scenarios include bowel obstruction due to peritoneal carcinomatosis, cytopenia due to bone marrow infiltration, hypercalcemia of malignancy, superior vena cava syndrome and cardiac tamponade. These situations are classified as oncologic emergencies and often require additional specific interventions beyond systemic oncologic therapies, potentially influencing clinical outcomes [5].

The estimated incidence of VC is up to 15% at the initial presentation of metastatic breast cancer (MBC), potentially rising to 60% in patients with visceral metastases [6] during the disease course. BC-related VC is generally associated with a poor prognosis, despite appropriate therapy [4, 5, 7]. Treatment recommendations for metastatic BC with VC are based on limited evidence, as this patient population has typically been excluded from major clinical trials. Classically, combination chemotherapy (CT) is recommended for the treatment of VC, based on higher response rates and more rapid time to response with this regimen [8, 9]. However, this paradigm has been recently questioned with the established role of cyclin-dependent kinase 4/6 (CDK4/6) inhibitors in treating hormone-receptor positive/HER-2 negative (HR+/HER2-) metastatic disease [10–13], in addition to other emerging innovative therapies such as antibody-drug conjugates (ADCs).

Despite therapeutic advances in metastatic BC, the inclusion of patients with VC in clinical trials remains an unmet need. Furthermore, access to innovative therapies is limited, particularly in low- and middle-income countries (LMICs), where cytotoxic CT remains the mainstay of treatment [14]. There are few available data evaluating and validating, in a real-world setting, the expert recommendation of polychemotherapy for the management of MBC in VC. This study aims to assess survival outcomes and explore prognostic factors of palliative CT for VC due to BC in a real-world scenario.

## Patients and methods

### Study design and population

A retrospective, observational, single-center study was conducted at a public cancer center in Brazil (Instituto do Câncer do Estado de São Paulo, University of São Paulo), collecting data from patients with BC hospitalised between 2008 and 2022.

We included patients diagnosed with MBC presenting with VC, based on an expanded definition considering the 5th ESO-ESMO International Consensus Guidelines for Advanced Breast Cancer (ABC5) [15]. According to ABC5, the hepatic VC was defined as elevated bilirubin > 1.5 times the upper limit of normal; pulmonary VC was characterised by resting dyspnea unrelieved by pleural effusion drainage. Impending liver VC was identified as a large volume of hepatic metastasis with elevated transaminases, without concurrent bilirubin elevation; while impending lung VC was defined as a large volume of pulmonary disease without oxygen desaturation or significant resting dyspnea [15]. Although not explicitly defined in the ABC5 guidelines, patients with cytopenia secondary to bone marrow infiltration or malignant bowel obstruction due to peritoneal carcinomatosis were also included, as these are conventionally considered VC scenarios in previously published studies [5].

Patient screening in electronic medical records was conducted through a search of keywords related to VC, such as: VC, liver failure, lymphangitis, bone marrow infiltration and bowel obstruction. Additionally, we filtered patients with VC among those who received CT during their hospitalisation. Some patients were screened during hospitalisation, but had experienced a VC or impending VC prior to the index hospital admission or were treated in an outpatient setting, and these cases were also included in the analysis.

Exclusion criteria were as follows: patients not meeting the aforementioned VC criteria or those with organ failure likely secondary to other underlying clinical conditions (e.g., sepsis, heart failure, pulmonary embolism and acute-on-chronic liver failure); symptomatic central nervous system metastases; >3 prior lines of CT in the metastatic setting or the presence of a concomitant active second malignancy.

### Objectives

The primary objective of the study was to evaluate survival outcomes associated with treatment for VC in MBC, as well as to identify potential prognostic factors associated with survival. Additionally, we aimed to describe treatment patterns, hospitalisation outcomes and the incidence of CT administration during the last month of life in this population.

### Data collection and statistical analysis

Data were collected using a REDCap case report form. Missing data were handled using the deletion method. Baseline characteristics, treatment patterns and hospitalisation outcomes were presented using descriptive statistics, with categorical variables described as relative and absolute frequencies and continuous variables as median and range. Survival analyses were performed using the Kaplan–Meier method. Prognostic factors associated with survival were evaluated through univariate and multivariable analyses using the Cox regression model. Statistical significance was defined as a *p*-value < 0.05. All statistical analyses were performed using Stata software, version 15.1 (StataCorp, Texas, USA).

### Ethical considerations

The study adhered to the ethical guidelines of the Declaration of Helsinki. Written informed consent was obtained from participants who could be located, and a waiver of consent was granted by the Institutional Review Boards for participants who could not be located or were deceased at the time of data collection.

## Results

### Baseline characteristics of the population

**Supplemental Figure 1.**
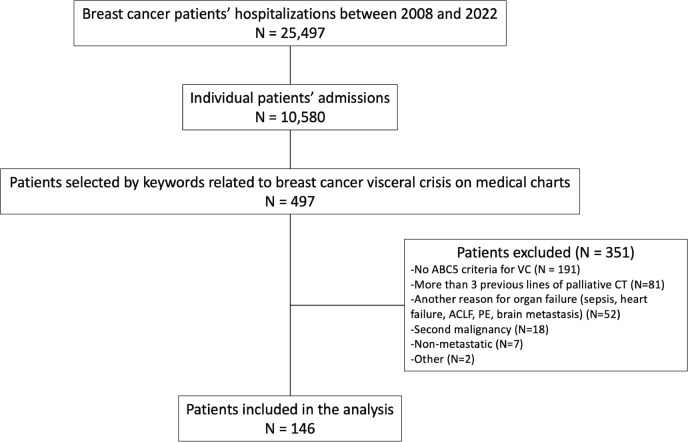
Patients’ selection flowchart. Abbreviations: ABC5, 5th ESO-ESMO international consensus guidelines for ABC; ACLF, acute on chronic liver failure; CT, chemotherapy; PE, pulmonary embolism; VC, visceral crisis.

Between 2008 and 2022, there were 25,497 hospital admissions for BC at our institution, encompassing 10,580 individual patient admissions. Searching for keywords related to VC in medical charts, 497 patients were selected, of whom 351 met exclusion criteria. Consequently, 146 patients with VC were included in the final analysis (Supplemental Figure 1).

The median age of the patients was 46.2 years (range 24–86), 63% had HR-positive/ HER2−negative BC, 24% had HER2-positive disease and 11% had triple-negative breast cancer (TNBC). Most of the patients had recurrent disease (56%), while 43% had *de novo* metastatic disease. The predominant type of VC was pulmonary (36.3%), followed by hepatic (32.2%) and bone marrow infiltration (19.2%). Impending pulmonary and hepatic VC accounted for 5.5% and 4.8% of cases, respectively (Table 1).

Nearly half of the patients (47.3%) had previously received (neo)adjuvant CT, and 32.2% had undergone palliative endocrine therapy (ET) before the diagnosis of VC. At the time of VC, 65.1% of the patients had not received any lines of palliative CT, while 15.8% had received one CT line and 17.8% had received two or more lines prior to the diagnosis of VC (Table 1).

### Types of therapy and outcomes of hospitalisation for VC

VC management was primarily based on combination CT (51.4%), while 27.4% were treated with single-agent CT and 20.6% received best supportive care (BSC) only. Among the 30 patients who received BSC only, the median age was 46 years, 76.67% had ECOG-PS 3 or 4 at the time of VC diagnosis, 46.67% had a hepatic VC and 90% had received at least one previous line of treatment.

The most frequently used chemotherapeutic agents were paclitaxel (34.3%), gemcitabine (24%), cisplatin (24%), carboplatin (14.4%) and doxorubicin (6.9%). The predominant combinations among the patients who received polychemotherapy (*N* = 67) were cisplatin+gemcitabine (33 patients, 49.25% of those receiving polychemotherapy), carboplatin+paclitaxel (18 patients, 26.87%) and doxorubicin+cyclophosphamide (6 patients, 8.96%) (Supplemental Table 1).

The main outcomes following hospitalisation for VC were death during the same hospitalisation (45.2%) and discharge with partial resolution of VC (37%). Most patients died in the ward, while approximately 30% died in the intensive care unit. The incidence of CT administration during the last month of life was 43.8% among patients with VC.

The relationship between the treatment modality and the type of VC is illustrated in Supplemental Table 2. Among patients with hepatic and pulmonary VC, nearly 60% received treatment with polychemotherapy. In contrast, for patients with cytopenia due to bone marrow infiltration, the majority received monochemotherapy (63%) and 15% received polychemotherapy.

### Survival outcomes

**Figure 1.**
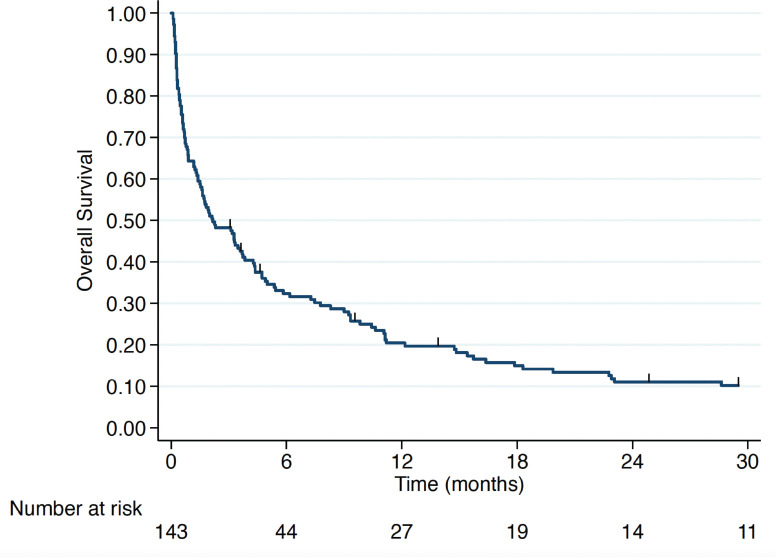
Overall survival of all VC patients.

**Figure 2.**
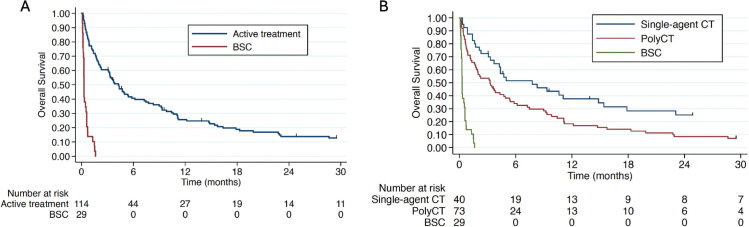
Overall survival according to the modality of treatment, comparing BSC versus any type of CT (a) or versus polychemotherapy and single agent CT (b). BSC: best supportive care; CT: chemotherapy.

After a median follow-up of 2 months, among the 146 patients with VC, 143 had survival data available, with 135 deaths recorded during the study period. At the close of data collection, the median overall survival (mOS) for the entire population was 2.17 months (Figure 1). Patients who received any form of active treatment had a mOS of 4.27 months (IQR 1.23–12.17), with 7.77 months (IQR 2.13–37.2) for single-agent CT and 3.27 months (IQR 0.83–9.83) for polychemotherapy. Patients who received the BSC alone had a mOS of 0.3 months (IQR 0.2–0.6) (Figure 2).

**Figure 3.**
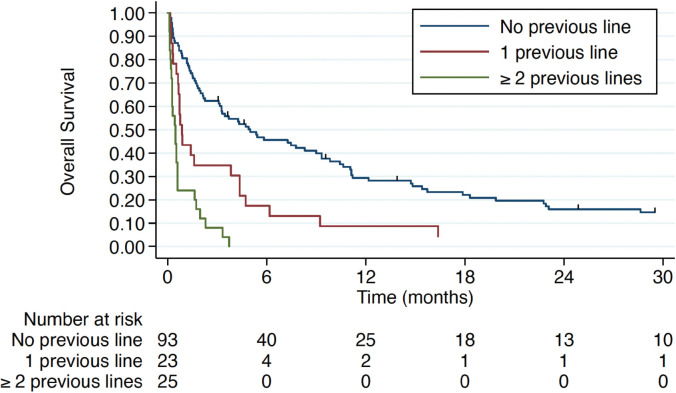
Overall survival according to the therapy line.

**Figure 4.**
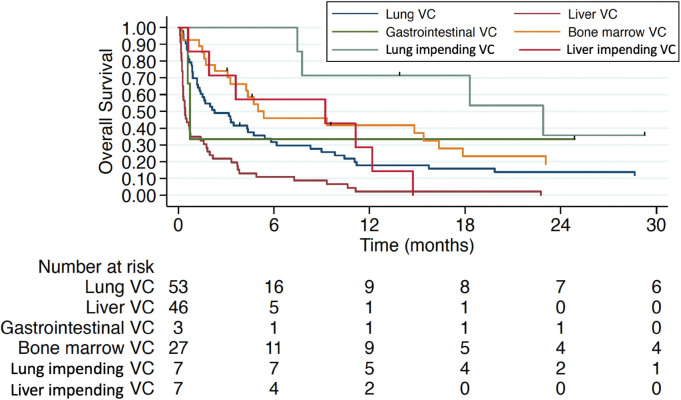
Overall survival in different types of VC. VC: visceral crisis.

Treatment for VC in the first-line setting was associated with a mOS of 5 months (IQR 1.4–15.4) compared to 0.87 months (IQR 0.53–4.37) and 0.47 months (IQR 0.23–0.6) in patients who had received one or ≥2 prior lines of CT in the advanced setting, respectively (Figure 3). Hepatic and pulmonary VC were associated with worse overall survival, as illustrated in Figure 4. Patients with TNBC had the poorest overall survival (0.9 months, IQR 0.6–1.77), compared to 3.47 months (IQR 0.63–15.4) in HR+ patients and 3.17 months (IQR 0.6–7.27) in HER2+ patients.

### Univariate and multivariable cox regression of factors associated with mortality

In the univariate Cox regression analysis, the HER2+ and TNBC subtypes were associated with higher mortality compared to HR+ patients. Additionally, hepatic VC, ≥1 prior line of CT before VC diagnosis and the absence of active oncological therapy (BSC only) were significantly associated with worse overall survival. Polychemotherapy, as compared to single-agent CT, also emerged as a risk factor for mortality in the univariate analysis. In the multivariate analysis, only hepatic VC and the absence of active oncological therapy (BSC only) maintained statistical significance as risk factors for mortality (Table 2).

## Discussion

This study is one of the largest cohorts with real-world data on the treatment of MBC in VC. In our study, the main type of VC was pulmonary (36%), followed by hepatic (32%). This finding aligns with other cohorts that have also reported liver and lung VC as the most common subtypes [6, 16, 17]. There is significant heterogeneity in the definition of hepatic and pulmonary VC across different studies. Some authors [6, 16, 17] defined hepatic VC solely as an increase in transaminases, without necessarily an increase in bilirubin levels, which would be considered an impending VC according to ABC5 criteria [15]. For the definition of pulmonary VC, most studies consider dyspnea in the presence of lymphangitic carcinomatosis or a high pulmonary tumour burden, although ‘requirement for thoracentesis’ was considered an inclusion criterion in one study [16], which does not reflect the definition present in the last ABC consensus [4].

Our results showed poor outcomes for patients in VC (mOS 2.17 months), even for those receiving treatment for VC in the first-line setting (mOS 5 months). This poor prognosis is illustrated by hospitalisation outcomes, where 45% of patients progressed to death. The incidence of CT administration in the last month of life was 43.8%, close to what was reported in other studies examining patients with MBC in VC, ranging between 42.6% and 65% [6, 17]. A population-based study from two European countries with patients with metastatic BC (not necessarily in VC) reported CT near the end-of-life at an incidence of 23.2% in a Swedish cohort and 46.5% in a Greek cohort [18]. CT use closer to the end of life is a known marker of poor-quality care and should be avoided [19], and our results and similar cohorts underscore the challenge of accurately determining the prognosis of this population.

The survival reported in other cohorts that included patients treated for VC varies from 4.7 weeks [17] to 11.2 months [20]. This variability arises from the fact that the concept of ‘VC’ encompasses multiple different clinical conditions with distinct prognoses. The cohort by Yang *et al* [20] which reported a general mOS of 11.2 months (6.2 months for those treated with CT), found a mOS of 8.1 months for liver VC and 18 months for bone marrow infiltration [20]. Besides the subtype of VC, other unfavorable prognostic factors reported in previous studies include a higher number of prior treatment lines, lack of VC resolution after CT, poor ECOG-PS, elevated bilirubin and high lactate dehydrogenase levels [6, 16, 20].

In our cohort, patients who received the BSC exclusively had worse overall survival (mOS 0.3 months). Although BSC remained a risk factor for mortality even in multivariate logistic regression, we believe this result may be related to a selection bias toward more severely ill patients who were not eligible for active oncological treatment. Among the 30 patients in our cohort who received BSC only at the time of VC diagnosis, 76.67% had ECOG-PS 3 or 4, 46.67% had a hepatic VC and 90% had received at least one previous line of treatment. The clinical decision regarding which patients are eligible for oncological treatment, particularly CT, must be highly judicious, considering the high rate of CT use in the last month of life.

Polychemotherapy also emerged as a statistically significant factor associated with higher mortality in univariate regression. This association may stem from the fact that patients undergoing polychemotherapy had a more severe type of VC. In our sample, patients with hepatic VC (subtype with the worst prognosis) were predominantly treated with polychemotherapy (59.6%). On the other hand, those with bone marrow infiltration were mostly treated with monochemotherapy (63%). Indeed, in the multivariable regression, the impact of polychemotherapy versus single-agent CT did not remain significant.

The distribution of BC subtypes in our sample closely reflected that of the general population, with 63% HR+/HER2−, 24% HER2-positive and 11% TNBC. Considering the selection of patients with VC, one might expect a predominance of more aggressive subtypes, such as triple-negative BC. However, this was not observed in our cohort. While TNBC patients had a relatively poor prognosis (mOS 0.9 months) as expected, HR+/HER2- and HER2-positive patients exhibited similar mOS (3.47 and 3.17 months, respectively). Real-world data for MBC patients (regardless of VC status) suggested superior OS for HER2-positive patients compared to HR+/HER2- and TNBC subtypes [21]. However, this distinction was not evident in our cohort. Even the eight HER2-positive patients who received anti-HER2 targeted therapy during the VC had an mOS of 3.8 months. This finding may relate to the delayed availability of anti-HER2 therapy within the Brazilian public healthcare system (trastuzumab available since 2011 and pertuzumab since 2020), as well as the lack of access to other anti-HER2 therapies in subsequent lines (e.g., trastuzumab emtansine and trastuzumab-deruxtecan (T-DXd)).

The paradigm of treating VC with polychemotherapy was recently questioned with the publication of the randomised phase II trial RIGHT CHOICE. This study included pre/perimenopausal patients with clinically aggressive HR+HER2- BC, of whom approximately 50% met the criteria for the VC. Patients were randomised to receive ET plus ovarian suppression and ribociclib or the investigator’s choice of polychemotherapy. There was a statistically significant improvement in progression-free survival (21.8 versus 12.8 months), without a difference in mOS (30-month OS of 66.6% versus 64.6%) and fewer adverse events with ET plus ribociclib. The median time to respond was 3.2 months with combination CT versus 4.9 months with ribociclib, although this difference was not statistically significant. However, the validity of these findings for patients with VC may be limited, as the PFS benefit was not observed for those with actual VC criteria in the subgroup analysis. Moreover, the study did not include patients with hepatic VC according to ABC criteria, as bilirubin >1.5x ULN was an exclusion criterion [13]. Indeed, the worse survival outcomes in our cohort compared to the RIGHT CHOICE trial highlight the more severe profile of hospitalised patients with the VC in our study compared to the patients included in that clinical trial.

Frontline combination of CDK4/6 inhibitor and ET for patients with aggressive HR+/HER2- BC was also evaluated in the ABIGAIL trial, a phase 2, non-inferiority study. Patients were randomised to receive abemaciclib plus ET (letrozole or fulvestrant) or paclitaxel for 12 weeks followed by abemaciclib plus ET. The 12-week overall response rate was 58.8% in patients treated with abemaciclib plus ET compared to 40.2% in patients treated with CT. The inclusion criteria of ‘aggressive HR+/HER2- BC’ was broad and the proportion of patients with the VC was not reported [22].

ADCs, such as T-DXd, have demonstrated promising efficacy with high objective response rates, surpassing those of conventional CT. In HER2+ patients, T-DXd has shown an objective response rate of approximately 80% in the second-line setting and near 50% in patients with

HER2-low expression after prior CT [23, 24]. While no trials have specifically addressed the VC scenario, the high response rates observed with ADCs suggest potential applicability in this context. Future trials should explore the role of ADCs in VC, although the challenges of including these patients in clinical trials must be acknowledged, considering also the fact that the applicability of such therapies is likely to vary depending on the BC subtype.

CDK4/6 inhibitors, ADCs, immunotherapy and PARP inhibitors are not available in the public healthcare system in Brazil. Therefore, our population’s treatment was based on cytotoxic CT, which also reflects the reality in most LMICs [14]. This recommendation is also endorsed by the ASCO resource–stratified guideline for systemic treatment of patients with MBC [25]. Furthermore, although recent evidence may suggest a role for these innovative therapies in managing VC [13, 26], the prevailing recommendation in guidelines for most of the time our cohort was treated was the use of polychemotherapy, which indeed was the predominant treatment choice in our sample. Considering the potential benefit of CDK4/6 inhibitors in clinically aggressive HR+HER2- BC, ensuring access to this therapy in LMICs should be a priority. Nevertheless, since the RIGHT CHOICE trial did not include severely ill patients, such as those with hepatic VC, polychemotherapy may still be an appropriate option for selected patients with HR+HER2- BC.

In our study, the incidence of VC was lower than in other cohorts, which may be associated with our strict selection criteria, including only patients with VC per the definitions established in the ABC5 guideline [15] or those with bone marrow infiltration and malignant bowel obstruction. Moreover, there was likely underdiagnosis in our cohort due to limitations in our inclusion method, which was based on the search for keywords related to VC in electronic medical charts of hospitalised patients. Indeed, 88.4% of our sample comprised patients who were hospitalised for treatment of VC. The screening method based on hospitalised patients introduces a selection bias towards more severe cases and leads to an underrepresentation of patients with impending VC, who may have received treatment in the outpatient scenario. Besides the inherent biases of a retrospective analysis, another limitation of our study was the lack of reporting treatment-related adverse events. During the 15-year period of our retrospective cohort (2008–2022), there was heterogeneous reporting in electronic medical records, with no standardisation or objective description of adverse events, which hindered this assessment.

## Conclusion

In conclusion, patients with MBC in VC have a poor prognosis even when treated with polychemotherapy. Factors such as the line of treatment, BC subtype, performance status and type of VC should be considered when discussing expected outcomes for this life-threatening condition. Proper prognostication of these patients is crucial for identifying those who may benefit from active systemic therapy while carefully avoiding potentially futile strategies. Although the inclusion of patients experiencing a VC in prospective trials is challenging, this could help to elucidate the efficacy and safety of CT and other emerging therapies in this scenario.

## Conflicts of interest

L.T.: Speaker fees and/or honoraria for consulting or advisory functions: Daiichi-Sankyo, MSD, AstraZeneca, Pfizer, Lilly, Novartis, Roche, Pfizer. Financial support for educational programs and symposia: AstraZeneca, Roche, Gilead, MSD. Institutional Research grant: Novartis. R.C.B.: Speaker fees and/or honoraria for consulting or advisory functions: Daiichi-Sankyo, Nestle Health Science, Addium, Gilead, MSD, BMS, AstraZeneca, Ache, Pfizer. Financial support for educational programs and symposia: AstraZeneca, Daiichi-Sankyo, MSD, Lilly. Institutional Research grant: Novartis, AstraZeneca. M.O.A. and V.H.F. declare no conflict of interest.

## Funding

This research did not receive any funding.

## Author contributions

M.O.A. contributed to the conceptualisation, data collection, writing and revision. V.H.F. contributed to the data collection, writing and revision.

L.T. contributed to writing and supervision. R.C.B. contributed to the conceptualisation, formal analysis, writing, revision and supervision.

## Figures and Tables

**Table 1.** Baseline characteristics of the population (N = 146).

Patient characteristic	*N* (%)
Age at VC diagnosis (median, range)	46.2 (24.1–86.2)
Age groups (at VC diagnosis)	
<40	38 (26.02%)
40–70	101 (69.17%)
>70	7 (4.79%)
Race	
White	88 (60.3%)
Mixed race	32 (21.9%)
Black	13 (8.9%)
Not reported	13 (8.9%)
Histology	
Ductal	130 (89.04%)
Lobular	11 (7.53%)
Other	5 (3.42%)
BC subtype	
HR+HER2-	92 (63.01%)
HR+HER2+	26 (17.81%)
HR-HER2+	9 (6.16%)
Triple negative	16 (10.95%)
Unknown	3 (2.05%)
Histological grade	
1	15 (10.27%)
2	62 (42.47%)
3	45 (30.82%)
Unknown	24 (16.44%)
Ki-67 (median, range)	40 (0–95)
Ki-67 groups	
≤15%	19 (13.01%)
>15%	102 (69.86%)
Unknown	25 (17.12%)
Anatomical staging at BC diagnosis	
I	6 (4.11%)
II	18 (12.33%)
III	53 (36.30%)
IV	63 (43.15%)
Unknown	6 (4.11%)
ECOG-PS (at VC diagnosis)	
1	50 (34.24%)
2	45 (30.82%)
3	29 (19.86%)
4	21 (14.38%)
Unknown	1 (0.68%)
Type of metastatic disease	
De novo	64 (43.84%)
Relapsed	82 (56.16%)
Sites of metastatic disease	
Bone	88 (60.27%)
Lymph nodes	40 (27.40%)
Lung	75 (51.37%)
Pleura	23 (15.75%)
Liver	65 (44.52%)
Peritoneum	3 (2.05%)
Central nervous system	7 (4.79%)
Other	9 (6.16%)
VC type	
Pulmonary VC	53 (36.30%)
Hepatic VC	47 (32.19%)
Bone marrow invasion	28 (19.18%)
Impending pulmonary VC	8 (5.48%)
Impending hepatic VC	7 (4.79%)
Malignant bowel obstruction (due to peritoneal carcinomatosis)	3 (1.89%)
Oncological treatment prior to VC	
None	38 (26.03%)
Adjuvant/neoadjuvant ET	56 (38.36%)
Adjuvant/neoadjuvant CT	69 (47.26%)
Adjuvant/neoadjuvant anti-Her2 therapy	5 (3.42%)
Adjuvant CDK4/6i	1 (0.68%)
Palliative ET	47 (32.19%)
Palliative CT	49 (33.56%)
Palliative anti-HER2 therapy	9 (6.16%)
Number of palliative CT lines prior to VC	
None	95 (65.07%)
1	23 (15.75%)
≥2	26 (17.8%)
Unknown	2 (1.37%)

Abbreviations: CDK4/6i, cyclin-dependent kinase 4/6 inhibitors; ECOG-PS, Eastern Cooperative Oncology Group Performance Status; HR, hormone receptor; VC, visceral crisis

**Table 2.** Univariate and multivariable cox regression of factors associated with mortality (*N* = 146).

Baseline factors	Univariate analysis			Multivariable analysis		
	HR	95% CI	*p*-value	HR	95% CI	*p*-value
BC subtype						
HR+HER2-	Reference					
HER2+	1.54	1.01–2.34	0.043	0.94	0.57–1.55	0.827
TNBC	2.60	1.47–4.59	0.001	1.32	0.69–2.54	0.391
Type of metastatic disease						
De novo	Reference					
Relapsed	1.17	0.83–1.67	0.353	-	-	-
VC type						
Pulmonary VC	Reference					
Hepatic VC	2.68	1.76–4.08	<0.001	3.45	2.15–5.55	<0.001
Malignant bowel obstruction	0.75	0.18–3.10	0.694	0.55	0.13–2.36	0.418
Bone marrow invasion	0.72	0.44–1.18	0.201	0.86	0.50–1.50	0.602
Impending pulmonary VC	0.37	0.15–0.95	0.040	0.54	0.21–1.40	0.206
Impending hepatic VC	1.00	0.44–2.22	0.999	1.59	0.66–3.82	0.299
ECOG-PS						
1–2	Reference					
3–4	6.74	0.91–49.65	0.061	4.17	0.50–34.55	0.185
Type of treatment						
Active treatment	Reference					
BSC	9.31	5.49–15.77	<0.001	-	-	-
Type of CT						
Monochemotherapy	Reference					
Polychemotherapy	1.59	1.05–2.41	0.026	1.20	0.73–1.99	0.460
BSC	12.65	6.9–23.18	<0.001	9.66	4.81–19.39	<0.001
Number of palliative CT lines prior to VC						
None	Reference					
1	2.18	1.36–3.50	0.001	-	-	-
≥2	5.70	3.44–9.44	<0.001	-	-	-
Age						
<60	Reference					
≥60	1.23	0.78–1.96	0.360	-	-	-
Ki-67						
<15	Reference					
≥15	1.39	0.83–2.30	0.202	-	-	-

CI: confidence interval; ECOG-PS: Eastern Cooperative Oncology Group Performance Status; HR: hazard ratio; VC: visceral crisis; TNBC: triple-negative breast cancer

## Supplementary tables and figure

**Supplemental Table 1.** Type of therapy and outcomes of hospitalisation for VC.

Therapy for VC	
Polychemotherapy	67 (45.89%)
Monochemotherapy	40 (27.40%)
CT + Anti-Her2	8 (5.48%)
ET only	1 (0.68%)
BSC	30 (20.55%)
Type of cancer therapy for VC	
Paclitaxel	50 (34.25%)
Gemcitabine	35 (23.97%)
Cisplatin	35 (23.97%)
Carboplatin	21 (14.38%)
Doxorubicin	10 (6.85%)
Cyclophosphamide	8 (5.48%)
Docetaxel	8 (5.48%)
Trastuzumab	8 (5.48%)
Capecitabine	4 (2.74%)
5-Fluorouracil	3 (2.05%)
Vinorelbine	1 (0.68%)
Pertuzumab	1 (0.68%)
Combination regimens (polychemotherapy)	
Cisplatin+gemcitabine	33 (22.60%)
Carboplatin+paclitaxel	18 (12.33%)
Doxorubicin+cyclophosphamide	6 (4.11%)
Doxorubicin+paclitaxel	3 (2.05%)
Other	7 (4.79%)
Outcome of hospitalization	
Discharge with total resolution of VC	7 (4.79%)
Discharge with partial resolution of VC	54 (36.99%)
Death	66 (45.21%)
No hospitalization required	17 (11.64%)
Unknown	1 (0.68%)
Place of death	
Ward	91 (62.33%)
Intensive care unity	43 (29.45%)
Emergency room	1 (0.68%)
Home	2 (1.37%)
Unknown	9 (6.16%)
CT during last month of life	
Yes	64 (43.84%)
No	73 (50.00%)
Unknown	9 (6.16%)

VC: visceral crisis

**Supplemental Table 2.** Treatment modality by type of VC – *N* (%).

	Monochemotherapy	Polychemotherapy	BSC
Pulmonary VC	14 (26.4%)	31 (58.5%)	8 (15.1%)
Hepatic VC	5 (10.6%)	28 (59.6%)	14 (29.8%)
Malignant bowel obstruction	1 (33.3%)	0	2 (66.7%)
Bone marrow invasion	17 (63%)	4 (14.8%)	6 (22.2%)
Impending pulmonary VC	3 (37.5%)	5 (62.5%)	0
Impending hepatic VC	0	7 (100%)	0

VC: visceral crisis
